# Pelvic Lymph Node Dissection Before Versus After Radical Cystectomy: A Systematic Review and Meta-Analysis

**DOI:** 10.1590/S1677-5538.IBJU.2024.0490

**Published:** 2024-01-13

**Authors:** Guilherme Melchior Maia Lopes, Luiz Guilherme Serrão Gimenez, Diogo Souto Santana, Rafael Baldissera Cardoso, Breno Cordeiro Porto, Rodrigo Afonso da Silva Sardenberg, Carlo Camargo Passerotti, José Pinhata Otoch, José Arnaldo Shiomi da Cruz

**Affiliations:** 1 Centro Universitário Faculdade de Medicina do ABC Santo André SP Brasil Centro Universitário Faculdade de Medicina do ABC (FMABC), Santo André, SP, Brasil; 2 Universidade de São Paulo Laboratório de Técnica Cirúrgica e Cirurgia Experimental Faculdade de Medicina São Paulo SP Brasil Laboratório de Técnica Cirúrgica e Cirurgia Experimental Faculdade de Medicina, Universidade de São Paulo – USP, São Paulo, SP, Brasil; 3 Hospital Moinhos de Vento Porto Alegre RS Brasil Hospital Moinhos de Vento, Porto Alegre, RS, Brasil; 4 Instituto Internacional de Ensino e Pesquisa Hapvida NotreDame Intermédica São Paulo SP Brasil Instituto Internacional de Ensino e Pesquisa - Hapvida NotreDame Intermédica, São Paulo, SP, Brasil

**Keywords:** Lymph Node Excision, Cystectomy, Urinary Bladder Neoplasms, Systematic Review [Publication Type]

## Abstract

**Purpose:**

Radical cystectomy (RC) is the standard of care for patients with bladder cancer, and pelvic lymph node dissection (PLND) is a pivotal step that can be carried out either before or after RC. Evidence on the optimal timing for PLND remains limited.

**Materials and Methods:**

We searched PubMed, Embase, Cochrane Central, Scopus and Google Scholar for studies comparing PLND before versus after RC. Outcomes assessed were total operative time, PLND time, RC time, number of lymph nodes (LN) dissected, and estimated blood loss. Mean differences (MDs) and 95% confidence intervals (CIs) were computed using a random-effects model. Subgroup analysis was conducted for robot-assisted RC (RARC).

**Results:**

A total of 801 patients from six studies were included, of whom 360 (44.94%) underwent PLND before RC. There were no significant differences in total operative time (MD −17.49; 95% CI −41.65,6.67; p = 0.16; I2 = 94%), PLND time (MD −14.91; 95% CI −44.91,15.09; p = 0.33; I2 = 96%), LN yielded (MD −1.13; 95% CI −4.81,2.55; p = 0.55; I2 = 83%), and estimated blood loss (MD 0.17; 95% CI −51.33,51.68; p = 0.99; I2 = 81%). However, RC time was significantly reduced (MD −28.89; 95% CI −42.84,-14.93; p < 0.0001; I2 = 75%) when PLND was performed prior to RC. In RARC studies, PLND before RC decreased total operative time, RC time, and estimated blood loss.

**Conclusions:**

The timing of lymphadenectomy was not associated with a significant reduction in total operative time, PLND time, LN yield, and estimated blood loss.

## INTRODUCTION

Bladder cancer (BCa) ranks as the nineth most frequently diagnosed malignant tumor worldwide, with over 60,000 new cases and more than 12,000 deaths reported annually among men in the United States (1, 2). Up to 40% of patients present with muscle-invasive bladder cancer (MIBC), and a quarter of them will harbor lymph nodal metastasis (3). Thus, early diagnosis and rapidly implemented interventions are essential in this type of tumor to reduce the risk of metastasis and improve survival rates. Radical cystectomy (RC) is currently regarded as the standard of care for patients with MIBC without systemic involvement, and also, though less frequently, for some non-muscle-invasive bladder (NMIBC) when intravesical treatments, such as BCG (Bacillus Calmette-Guerin), have failed (4, 5). RC is associated with a significant survival gain compared to observation, multiple resections, chemotherapy, or radiotherapy (6-8).

Pelvic lymph node dissection (PLND) is a pivotal stage of RC and can be carried out either before or after cystectomy. While current literature extensively discusses PLND templates, lymph node (LN) yield, density, positive pathological rates, and oncological benefits (9-11), there is limited evidence on the optimal timing of the procedure relative to RC, which is rarely addressed in guidelines. This uncertainty has raised concerns about potential impacts on perioperative outcomes, including operative time, blood loss, and postoperative recovery, which are critical for patient safety and long-term prognosis.

Furthermore, variability in clinical practices concerning the timing of PLND highlights the need for more concrete, evidence-based guidelines. Standardizing this component of RC could lead to improved consistency in outcomes across medical health centers and provide clearer instructions for urologists managing BCa cases. Therefore, we aimed to undertake a systematic review and meta-analysis to compare PLND performed before versus after RC to determine the optimal approach.

## MATERIALS AND METHODS

This systematic review and meta-analysis were performed and reported following the Cochrane Collaboration Handbook for Systematic Reviews of Interventions and the Preferred Reporting Items for Systematic Reviews and Meta-Analysis (PRISMA) Statement guidelines (12, 13). The prospective protocol was registered in the International Prospective Register of Systematic Reviews (PROSPERO; CRD42024550620)

### Eligibility criteria

Inclusion in this meta-analysis was restricted to studies that met all the following eligibility criteria: (I) randomized controlled trials (RCTs) or nonrandomized studies; (II) involving patients undergoing RC; (III) comparing PLND before versus after RC; and (IV) reporting any of the outcomes of interest. We excluded studies with (I) no control group; (II) no outcome of interest; (III) overlapping population; or (IV) preliminary results from published studies.

### Search strategy

We systematically searched PubMed (MEDLINE), Embase, Cochrane Central Register of Controlled Trials, Scopus, and Google Scholar from inception to June 2024. The search terms included ‘radical cystectomy’ and ‘lymphadenectomy’. No filters or language limitations were applied in our search. A complete electronic search strategy is reported in the Supplementary Appendix. After removing duplicates, two authors (G.M.M.L. and L.G.S.G.) screened the titles and abstracts and independently assessed full-text articles for inclusion based on prespecified criteria. Discrepancies were resolved in a discussion panel with the senior author. We also searched for additional eligible studies through a review of the references from articles identified in the original search.

### Data extraction

Two authors (G.M.M.L. and L.G.S.G.) independently extracted the data from each study using a standardized data collection document to collect the following characteristics: inclusion and exclusion criteria, total number of participants in each group, baseline characteristics, RC technique, pathological staging, pathological LN metastasis, limitations of each study, endpoint data, and endpoint definitions. Our prespecified primary endpoints were total operative time, PLND time, and RC time. Our secondary outcomes included the number of dissected LN, and estimated blood loss. Baseline characteristics were reported as the mean and standard deviation for continuous variables and proportion for binary variables.

### Quality assessment

We evaluated the risk of bias in randomized studies using version 2 of the Cochrane Risk of Bias assessment tool (RoB−2) (14), in which studies are scored as high, some concerns, low, or unclear risk of bias in 5 domains: selection, performance, detection, attrition, and reporting biases. Non-randomized studies were assessed with the Risk of Bias in Non-randomized Studies - of Interventions tool (ROBINS-I) (15). The two authors (G.M.M.L. and L.G.S.G.) independently conducted the assessments, and disagreements were resolved through consensus after discussing reasons for discrepancies.

### Statistical analysis

Endpoints were primarily analyzed with a mean difference (MD) with 95% confidence interval (CI). Cochran Q test and I2 statistics were used to assess heterogeneity. We used the DerSimonian and Laird random-effect model to calculate pooled estimates, considering that the patients came from different populations. Review Manager 5.4 (Cochrane Centre, The Cochrane Collaboration, Denmark) was used for statistical analyses.

## RESULTS

### Study selection and characteristics

**Figure 1:**
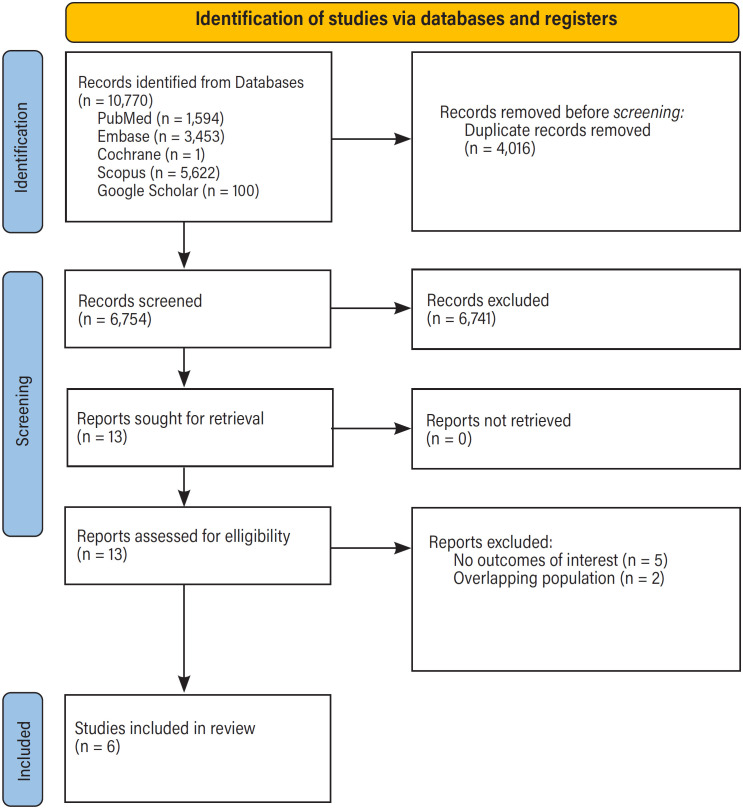
PRISMA flow diagram od study screening and selection.

Our initial search yielded 10,770 results, as shown in Figure-1. After removing duplicate records and ineligible studies, 13 were retrieved and remained for full-text revision based on our previously detailed inclusion criteria. Six studies were ultimately included in the pooled analysis, comprising 801 patients from one RCT (16) and five cohort studies (17-21). Among these patients, 360 (44.94%) underwent PLND before RC, whereas 441 (55.06%) underwent PLND after RC. The main characteristics of the included studies are presented in Table-1. The mean age of all patients included was 60.17 years old, with no significant difference between both groups, and 658 (82.15%) were male. The clinical and surgical baseline characteristics of the included patients are detailed in Table-2.

**Table 1:** Main characteristics of the included studies.

Study	Country; Period	Design	Exclusion criteria	RC technique
**Moeen, et al. 2024** (16)	Egypt; 2014-2019	RCT, single-center	Palliative cystectomy, grossly enlarged LNs in MSCT or MRI, CKD, or refused to participate	Open
**Kumaraswamy, et al. 2023** (17)	India; 2019-2022	Ambispective, single-center	Incomplete or missing data	Laparoscopic
**Wang, et al. 2023** (18)	China; 2014-2022	Retrospective, single-center	Previous bladder or prostate surgery, previous RT, distant metastasis, coagulation dysfunction, important organ dysfunctions, or combined with other systemic malignant tumors	RARC
**Salih Boga, et al. 2020** (19)	Turkey; 2017-2019	Retrospective, single-center	NA	RARC
**Zhu, et al. 2013** (20)	China; 2003-2013	Retrospective, single-center	Non-extended or zoned PLND, distant metastasis, or neoadjuvant RT or CR	RARC
**Ozen, et al. 2012** (21)	Turkey; 2005-2009	Prospective, multicenter	Previous pelvic RT, previous PLND, or neoadjuvant CT	Open

CKD = chronic kidney disease; CT = chemotherapy; LNs = lymph nodes; MRI = magnetic resonance imaging; MSCT = multi-sliced computed tomography; NA = not available; PLND = pelvic lymph node dissection; RARC = robot-assisted radical cystectomy; RC = radical cystectomy; RCT = randomized controlled trial; RT = radiotherapy

**Table 2:** Clinical and surgical baseline characteristics of the included patients.

Study	Patients, n		Age, years (±SD)			Male, n (%)			Pathological stage, n (%)									Pathologic LN metastasis, n (%)		
	bRC	aRC	bRC	aRC	p	bRC	aRC	p	bRC				aRC				p	bRC	aRC	p
									≤T1	T2	T3	T4	≤T1	T2	T3	T4				
Moeen, et al. 2024 (16)	83	86	58.21(±6.9)	55.03 (±7.6)	0.235	66 (79.5)	65(75.5)	0.326	0 (0)	50 (60.3)	33 (39.7)	0 (0)	0 (0)	52 (60.5)	34 (39.5)	0 (0)	0.513	13 (15.7)	18 (20.9)	0.111
Kumaraswamy, et al. 2023 (17)	22	22	57.95 (±12)	57.95 (±9.97)	1.0	19 (86)	22 (100)	0.23	6 (27.3)	11 (50)	5 (22.7)	0 (0)	7 (31.8)	12 (54.54)	2 (9.09)	1 (4.54)	0.62	NA	NA	1.0
Wang, et al. 2023 (18)	152	196	61.08 (±7.66)	62.75 (±5.753)	0.588	114(91.2)	157(80.1)	0.158	24(15.8)	56(36.8)	53(34.8)	19(12.5)	30(15.3)	62(31.6)	67(34.2)	37(18.9)	0.228	34(22.4)	53(27.1)	0.376
Salih Boga, et al. 2020 (19)	8	7	61.00(±7.67)	62.86(±5.98)	0.608	7(87.5)	7(100)	NA	0 (0)	5(62.5)	2(25.0)	1(12.5)	0 (0)	4(57.1)	2(28.6)	1(14.3)	0.978	3(37.5)	2(28.6)	0.714
Zhu, et al. 2013 (20)	47	60	63.00(±10)	61.00(±10)	NA	44(93.61)	50(83.33)	NA	1 (2.1)	12 (25.5)	21 (44.7)	13 (27.7)	2 (2.3)	16 (26.7)	29 (48.3)	13 (21.7)	NA	16 (34.0)	19 (31.7)	NA
Ozen, et al. 2012 (21)	48	70	61.09(±9.75)		NA	107(90.7)		NA	10(20.8)	12(25.0)	21(43.8)	5(10.4)	15(21.4)	20(28.6)	21(30.0)	14 (20.0)	0.181	19(39.6)	24(34.3)	0.385

aRC = After radical cystectomy; bRC = Before radical cystectomy; LN = lymph node; NA = Not Available; RC = Radical cystectomy; SD = Standard deviation.

### Pooled analysis of all studies

**Figure 2:**
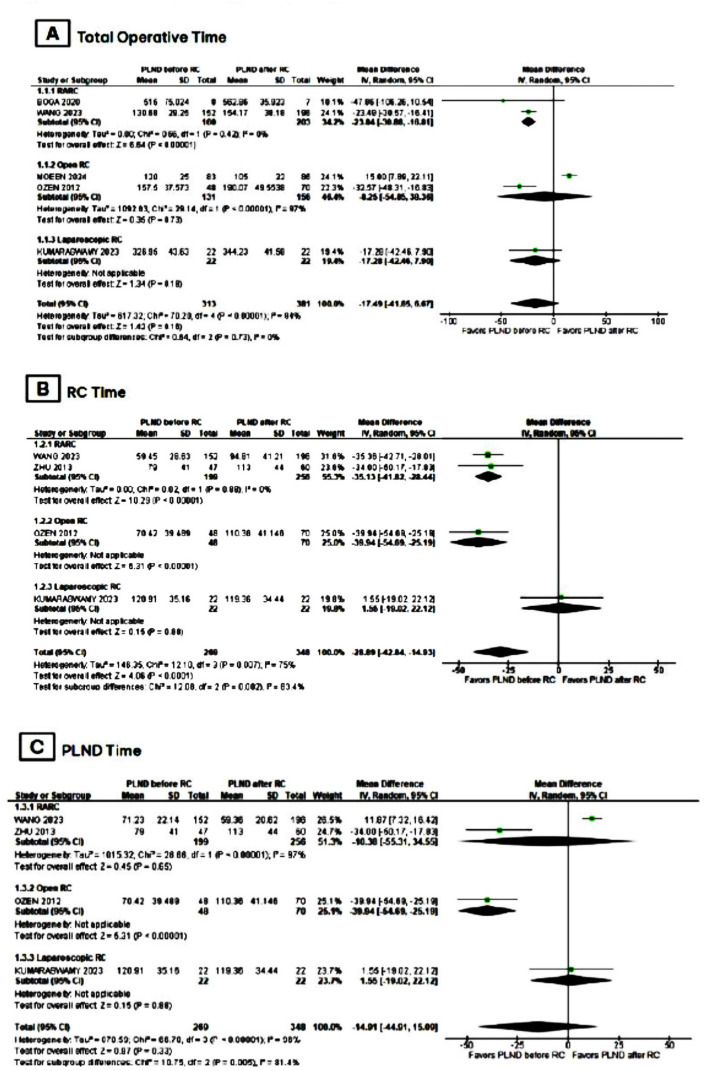
Meta-analysis of primary endpoints.

**Figure 3:**
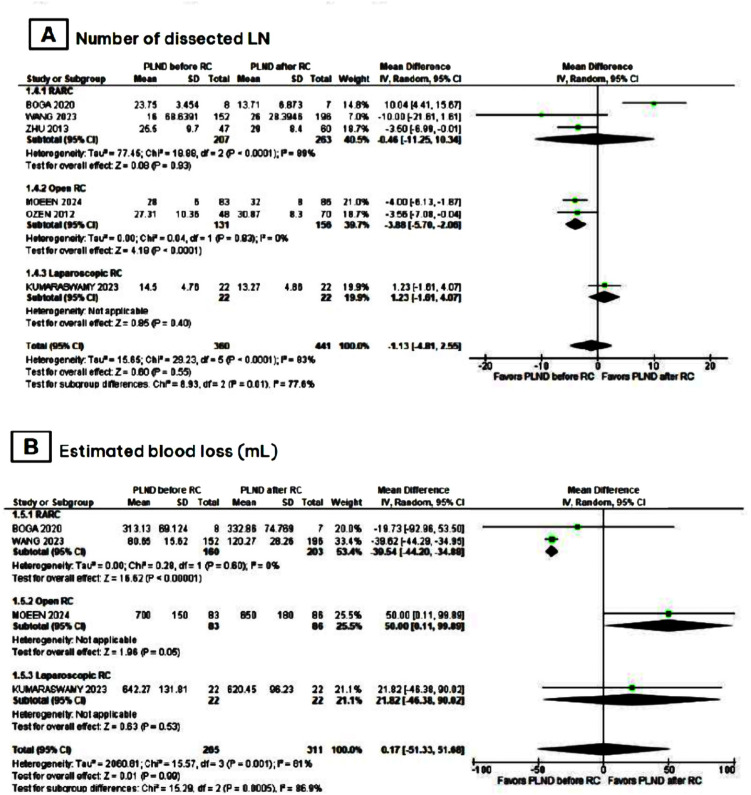
Meta-analysis of secondary endpoints.

In the group of patients that had PLND before RC, there was an overall trend towards decreased total operative time (MD −17.49; 95% CI −41.65,6.67; p = 0.16; I2 = 94%; Figure 2A) and significantly lower RC time (MD −28.89; 95% CI −42.84,-14.93; p < 0.0001; I2 = 75%; Figure-2B) when compared to those who underwent it after RC. Moreover, there was no statistical difference between both groups in PLND time (Figure-2C), number of LN dissected (Figure-3A), and estimated blood loss (Figure-3B).

(A) Recurrence rate; (B) Radical cystectomy time; (C) Pelvic lymph node dissection time.

CI = confidence interval; RARC = robot-assisted radical cystectomy; SD = standard deviation

(A) Number od dissected lymph nodes; (B) Estimated blood loos.

CI = confidence interval; RARC = robot-assisted radical cystectomy; SD = standard deviation

### Subgroup analysis

In a subgroup analysis of studies that performed robot-assisted RC (RARC), there was a significant reduction in total operative time (MD −23.84; 95% CI −30.88,-16.81; p <0.00001; I2 = 0%; Figure-2A), RC time (MD −35.13; 95% CI −41.82,-28.44; p < 0.00001, I2 = 0%; Figure-2B), and estimated blood loss (MD −39.54; 95% CI −44.20,-34.88; p <0.00001; I2 = 0%; Figure-3B) in patients that had PLND before RC. Furthermore, there was no statistical difference between groups in the number of LN dissected (Figure-3A).

### Quality assessment

Supplementary Appendix Figure-1 summarizes the individual risk of bias assessments of the included studies. The RCT was appraised using the Cochrane Collaboration's tool RoB-2, and it was considered to have an overall risk of bias classified as "some concerns", primarily due to the nature of the procedure, since it is inherently impossible to blind the surgeon. All five non-randomized studies were rated as "moderate risk" due to their potential to introduce confounding factors or bias in patient selection. Furthermore, the retrospective design of four of these studies might influence the determination of patient exclusion criteria based on specific findings such as outcomes and comorbidities.

## DISCUSSION

In this systematic review and meta-analysis comprising six studies and 801 non-overlapping patients, we comprehensively compared performing PLND before or after RC. The main findings from our pooled analysis did not demonstrate statistically significant differences in total operative time, PLND time, number of LN dissected, and estimated blood loss. However, there was a significant reduction in RC time in patients that underwent PLND before RC.

Lymph node involvement in BCa is a crucial prognostic factor for oncological outcomes, and its incidence ranges from 5% in NMBIC and 18-27% in MBIC. Given the heightened risk of postoperative tumor recurrence associated with nodal metastases, PLND is a pivotal component of RC (22, 23). Multiple aspects have been studied to contribute to a safe and effective PLND, such as the extent of the dissection, the number of LN yielded, and the surgical technique.

The lymphatic drainage in bladder cancer surgery can follow two main templates: a limited PLND, which includes both sides of the obturator fossa, and an extended PLND, which covers a broader area, such as the aortic bifurcation, iliac vessels, and internal iliac nodes (24, 25). Studies have shown that extended PLND is associated with better relapse-free survival (RFS) due to improved local control, though extending beyond this (super-extended PLND) does not improve survival and may increase complications (3, 26-27).

A higher number of lymph nodes (LNs) removed correlates with better survival rates, as it helps remove micrometastases and ensures more accurate staging (28-31). Research suggests that patients with at least 10 nodes removed tend to have better outcomes, and some recommend dissecting 15 to 20 nodes. However, rather than focusing solely on the number of nodes, the meticulous performance of the dissection within a well-defined template is more important for better oncological outcomes (32-34).

The optimal timing of PLND relative to RC has been controversial. Advocates for performing PLND before RC argue that this approach bares the vascular pedicles of the urinary bladder, which allows for easier identification and control of these blood vessels, potentially reducing the risk of significant blood loss and making the subsequent steps of cystectomy faster and more efficient. However, the narrow pelvic space, especially in patients with large or locally advanced tumors, may make the procedure more challenging. On the other hand, proponents of performing PLND after RC emphasize the advantages of a wider operative field in the narrow pelvic cavity once the bladder is removed. The expanded surgical field facilitates the procedure, particularly in cases where previous pelvic surgery or tri-modality treatments have resulted in marked pelvic adhesions (16, 17, 21). Our study demonstrated a statistically significant reduction in RC time in patients who underwent early PLND, yet it did not find significant superiority in performing PLND before or after RC regarding the total operative time, PLND time, number of LNs yielded, and estimated blood loss. Moreover, this issue is not addressed in the guidelines of international medical associations, such as the American Urological Association (AUA) and the European Association of Urology (EAU) (4, 5, 35, 36). Consequently, the timing of PLND should be based on the surgeon's experience and preference, as well as the patient-related factors, to provide an effective procedure with minimal morbidity.

In recent years, advancements in surgical technology have impacted the approach to RC for BCa treatment. Despite typically requiring more operative time than open RC, RARC offers substantial benefits, such as smaller incisions, reduced blood loss, earlier bowel motility, fewer postoperative complications, and quicker recovery times. This increased surgical duration might be attributed to the complex setup of the robotic system, the docking of the robot, and the learning curve associated with mastering robotic surgical techniques (37-40). Our study showed that patients who had robotic PLND before RARC presented a statistically significant reduction in total operative time, RC time, and estimated blood loss. Therefore, performing PLND before cystectomy appears to be a favorable option for patients undergoing the robotic procedure.

This study has some limitations. Firstly, the scarcity of available literature on the optimal timing of PLND has led to a relatively small sample size, impacting the depth and robustness of our analysis and potentially restricting the generalizability of our results. Secondly, the generalizability of our findings may be affected by a geographical limitation, given that studies from Europe or the United States, regions known for their significant contributions to oncological research, were either not available or did not meet the inclusion criteria. Additionally, we observed significant heterogeneity in the outcomes studied. This increased heterogeneity could stem from multiple factors across the included studies, such as variability in surgical techniques used for RC and PLND, differences in surgeons’ expertise, and inconsistencies in perioperative protocols. Moreover, patient-related variables, such as differences in tumor characteristics, baseline health status, and prior treatments, may further contribute to the observed heterogeneity, which underscores the need for more standardized protocols and reporting to reduce variability and improve comparability between studies. Lastly, there is a paucity of RCTs comparing PLND before and after RC, highlighting the importance of further research in this area.

## CONCLUSION

In this meta-analysis including 801 patients who had PLND performed before or after RC, the timing of the lymphadenectomy was not associated with a significant reduction in total operative time, PLND time, number of LN dissected, and estimated blood loss. Additional RCTs are required to assess the comparative effectiveness of PLND before versus after RC and the oncological outcomes.

## ABBREVIATIONS

BCa: = Bladder cancer
MIBC: = Muscle-invasive bladder cancer
RC: = Radical cystectomy
NMIBC: = Non-muscle-invasive bladder cancer
BCG: = Bacillus Calmette-Guerin
PLND: = Pelvic lymph node dissection
LN: = Lymph node
RCT: = Randomized controlled trial
MD: = Mean difference
CI: = Confidence interval
RARC: = Robot-assisted radical cystectomy
